# Sleep stage and obstructive apneaic epoch classification using single-lead ECG

**DOI:** 10.1186/1475-925X-9-39

**Published:** 2010-08-19

**Authors:** Bülent Yılmaz, Musa H Asyalı, Eren Arıkan, Sinan Yetkin, Fuat Özgen

**Affiliations:** 1 Faculty of Engineering, Electrical-Electronics Engineering Department, Zirve University, Gaziantep, Turkey; 2 Natural and Applied Sciences Institute, Biomedical Engineering Department, Başkent University, Ankara, Turkey; 3 Psychiatry Clinic, Military Hospital, Diyarbakır, Turkey; 4 Psychiatry Clinic, Gülhane Military Medical Academy (GATA), Ankara, Turkey

## Abstract

**Background:**

Polysomnography (PSG) is used to define physiological sleep and different physiological sleep stages, to assess sleep quality and diagnose many types of sleep disorders such as obstructive sleep apnea. However, PSG requires not only the connection of various sensors and electrodes to the subject but also spending the night in a bed that is different from the subject's own bed. This study is designed to investigate the feasibility of automatic classification of sleep stages and obstructive apneaic epochs using only the features derived from a single-lead electrocardiography (ECG) signal.

**Methods:**

For this purpose, PSG recordings (ECG included) were obtained during the night's sleep (mean duration 7 hours) of 17 subjects (5 men) with ages between 26 and 67. Based on these recordings, sleep experts performed sleep scoring for each subject. This study consisted of the following steps: (1) Visual inspection of ECG data corresponding to each 30-second epoch, and selection of epochs with relatively clean signals, (2) beat-to-beat interval (RR interval) computation using an R-peak detection algorithm, (3) feature extraction from RR interval values, and (4) classification of sleep stages (or obstructive apneaic periods) using one-versus-rest approach. The features used in the study were the median value, the difference between the 75 and 25 percentile values, and mean absolute deviations of the RR intervals computed for each epoch. The k-nearest-neighbor (kNN), quadratic discriminant analysis (QDA), and support vector machines (SVM) methods were used as the classification tools. In the testing procedure 10-fold cross-validation was employed.

**Results:**

QDA and SVM performed similarly well and significantly better than kNN for both sleep stage and apneaic epoch classification studies. The classification accuracy rates were between 80 and 90% for the stages other than non-rapid-eye-movement stage 2. The accuracies were 60 or 70% for that specific stage. In five obstructive sleep apnea (OSA) patients, the accurate apneaic epoch detection rates were over 89% for QDA and SVM.

**Conclusion:**

This study, in general, showed that RR-interval based classification, which requires only single-lead ECG, is feasible for sleep stage and apneaic epoch determination and can pave the road for a simple automatic classification system suitable for home-use.

## Introduction

Sleep is defined as the naturally recurring state of rest during which consciousness of the world is suspended [1]. Sleep is categorized into two types: Rapid Eye Movement (REM) and Non-Rapid Eye Movement (NREM). REM and NREM sleep alternate cyclically through the night. NREM sleep is further divided into four stages (NREM 1 to NREM 4) [2,3].

Sleep shows a complex, highly organized pattern of diverse physiological variables. Polysomnography (PSG) is used to define physiological sleep and the different physiological sleep stages, to diagnose many types of sleep disorders including narcolepsy, restless legs syndrome, REM behavior disorder, parasomnias, and sleep apnea [4]. A PSG system is typically placed in a sleep laboratory and includes a minimum of eleven channels, including electroencephalogram (EEG), electromyogram (EMG), electrooculogram (EOG), oxygen saturation (SpO2), and one channel electrocardiogram (ECG). Multi-channel data is simultaneously recorded both on continuously moving chart paper and to a computer system for analysis and displaying purposes [5]. 30-second epochs are the basic time periods on which data analysis and interpretation is performed. For special purposes, occasionally longer or shorter epochs are scored.

Typically, manual sleep stage classification is based on three data sources: EEG, EOG, and chin EMG [6]. Using this dataset, each epoch is scored as *wake*, *REM*, or one of the *NREM *stages. The limiting aspects of this type of data acquisition are the placement of EEG electrodes on the scalp, and the manual scoring.

Sleep apnea is a complete or near complete cessation of airflow for at least 10 seconds. There are three forms of sleep apnea: central, obstructive, and mixed [7]. Breathing is interrupted by the lack of respiratory effort in central sleep apnea; in obstructive sleep apnea, breathing is interrupted by a physical block to airflow despite respiratory effort. In mixed sleep apnea, there is a transition from central to obstructive features during the apnea events. Sleep apnea detection is performed by the overnight acquisition of ECG, airflow measurement, respiratory effort and SpO_2 _in addition to EEG, EOG, and EMG.

Hypopnea is defined as a reduction in the amplitude of the airflow of at least 50%, lasting at least 10 seconds, followed by either a decrease in SpO_2 _of 4%, or signs of physiological arousal [8].

Apnea/Hypopnea Index (AHI) or Respiratory Disturbance Index (RDI) is defined as the total number of apneas and hypopneas per hour. AHI or RDI are used to assess the quality of sleep. AHI values are typically categorized as 5-15 mild, 15-30 moderate, and above 30 listed as severe. If a 7-hour sleep (840 epochs), for example, includes a total number of epochs with apnea and hypopnea over 35 (i.e., approximately 4% of the epochs are apneaic), this subject is diagnosed as mild sleep apnea patient. For a severe apnea patient in 25% of the epochs apnea or hypopnea is observed.

Although sleep apnea is a respiratory event, it can affect the systems in the body, especially the cardiovascular system. Therefore, the ECG can provide very valuable information about apneaic events [9].

PSG recordings obtained in sleep laboratories require not only the connection of various sensors and electrodes to the subject but also spending the night in a bed that is different from the subject's own bed. One can argue that these requirements might affect the subject's sleep characteristics and a simpler acquisition system for home-use can be more efficient and accurate in assessing sleep quality. Along these lines, recently an at-home sleep analysis system (Alice PDx, Philips, Einthoven, The Netherlands) has been commercialized to help diagnose mainly the obstructive sleep apnea (OSA). This system measures patient airflow using nasal cannula and/or oral thermistor, and SpO_2_. Patients can administer the system in their home before going to sleep, and data is then transferred to a physician via a memory card.

Another system in which ECG electrodes (for power spectral analysis of the heart rate) combined with a miniature body movement sensor (a piezo film accelerometer) are attached to the chest was proposed for a simple and inexpensive differentiation between the wake, REM and NREM stages (U.S. Patent Number 5280791). In addition, a wristwatch, which monitors one's sleep cycle from wakefulness to REM by tracking a succession of body movements (using an accelerometer), has recently been available in the market (Sleep Tracker Pro, Innovative Sleep Solutions Inc., Atlanta, GA, USA).

Along with these commercial products and patents, in a recent study, Hamann and coworkers showed that the synchronization between the heartbeat and breathing pattern is significantly enhanced during certain stages of sleep. By mathematically analyzing heart rate throughout the night, they obtained information about breathing and sleep stages of subjects [10].

Finally, in the last years, a number of studies involved the use of single-lead ECG recordings (for example, Holter monitor data) for the detection of obstructive sleep apnea (OSA). In 2000, the aim of the "Computers in Cardiology Challenge" was to pave the way to the development of approaches for detecting and quantifying sleep apnea based on only ECG. Penzel et al. [11] reported the comparison of the methods used in the challenge and investigated how several of the most successful strategies can be combined. They reported that the algorithms made use of frequency-domain features to estimate changes in heart rate and the effect of respiration on the ECG waveform performed the best. Very recently, Mendez and coworkers [9] tested time-varying autoregresive model and k-nearest-neighbor method for the same purpose on Physionet sleep apnea dataset.

Since manual scoring is a subjective and time-consuming process, various automatic sleep staging and OSA detection studies have been carried out. They mainly involve four steps: i) Preprocessing of the data obtained using PSG or other acquisition systems (e.g. noise cancellation, removal of the highly noisy epochs), ii) feature extraction (e.g. power spectral density, R-peak detection, Wavelet Transform, time-frequency analysis), iii) supervised or unsupervised classification approaches for final decision, and iv) performance analysis by comparing the computer-generated results with the sleep experts' interpretation. Previously, automatic methods aiming the sole use of ECG [9,11,12], EOG [13], or EEG [14,15] have been investigated.

Because placement of multiple electrodes on the scalp is a tedious procedure and can properly be performed by experienced sleep technicians only, placement of three self-adhesive electrodes on the thorax for single-lead ECG recordings may be a better option for sleep staging at home. An ECG-based alternative would also be useful for OSA detection instead of nasal airflow sensor and SpO_2 _based approach. Thus, there is a need for simple automatic sleep analysis methods based on ECG recordings.

In this study, a single-lead ECG signal was used for automatic sleep stage classification and OSA detection. The features derived from solely the beat-to-beat intervals computed at each epoch served as the basis of the study. The computation of the features selected here did not require any spectral or wave shape analysis. Three well-known classification approaches, k-nearest-neighbor (kNN), quadratic discriminant analysis (QDA), and support vector machines (SVM) on the sleep ECG recordings from 8 healthy subjects and 9 OSA patients were investigated. The methods studied range from simple (kNN) to more complicated (SVM) classification approaches.

## Materials and Methods

### Study Population and Signal Acquisition

In this retrospective study we have worked on polysomnography data of 17 subjects (5 men). All data analyzed were collected as part of routine diagnosis and treatment, therefore we did not need an ethical committee approval. The data consisted of recordings of 32 channels of physiological parameters (Somno Star Alpha Series 4, Sensor Media Corporation, Yorba Linda, CA) at Gülhane Military Medical Academy, Psychiatry Clinic Sleep Laboratories, Ankara, Turkey. Along with the EEG, EOG and EMG recordings, signals from sensors for oronasal respiration, thoracic and abdominal movement, oxygen saturation, and electrical activity of the heart (ECG) were acquired. Sleep experts (the authors SY and FO) manually performed the sleep stage determination on the subjects using the PSG recordings. Each 30-second epoch was annotated as one of the four NREM stages, or wake, or REM stage. The experts also annotated each epoch whether there was an obstructive apneaic period (existence of OSA) or not. Because this study solely required single-lead ECG, the associated signal (lead II) was extracted from each subject's PSG recordings. The sampling frequency used for ECG acquisition was 200 Hz and the band-pass filter cut-off frequency values were set at 0.5 Hz and 40 Hz.

From 17 subjects enrolled in the study, 8 did not have any known health problems (healthy group), and 9 were previously diagnosed with obstructive sleep apnea (OSA group). The average age for the healthy group (1 man) was 27.2 (min. 26, max 34), and for the OSA group (4 men) it was 52.3 (min. 43, max. 67). The average sleep duration for the healthy group was 7.1 hours (min. 5.4, max. 8.4 hours), and for the OSA group it was 6.9 hours (min. 6.2, max. 7.3 hours). Table 1 shows the average percentage of the duration of each sleep stage for healthy and OSA group. In addition, in an average of 12.7% of the epochs, an apneaic event was detected. In our OSA group, the rate of epochs with apnea ranged from 5% to 25%, which represents different severity of the disease, from mild to severe. As depicted in Table 1, the mean Apnea/Hypopnea Index (AHI) for the OSA group was 21 (from 6.5 to 41.2).

**Table 1:** The average percentage values obtained for different sleep stages for healthy and OSA group.

	Wake %	NREM 1%	NREM 2%	NREM 3%	NREM 4%	REM %	AHI
Healthy	5.5	1.8	58.6	6.0	15.1	13.0	N/A

OSA	14.5	4.6	70.0	2.6	1.1	7.5	21

### ECG Preprocessing and Feature Extraction

A Matlab-based (The Mathworks Inc., Natick, MA, USA) custom ECG viewer software allowed the user to visually select the epochs to be included in the subsequent studies. The selection criteria were the noise level and number of arrhythmic beats present in the ECG data for a particular epoch. Contractions of heart ventricles produce relatively large potential deflections in the ECG signal, known as the R-peaks. The intervals between these peaks in the ECG are referred as beat-to-beat or RR intervals.

The subsequent analysis on the selected epochs was comprised of three steps: (1) beat-to-beat (RR) interval computation for each epoch, (2) feature extraction from RR-interval values for each epoch, and (3) classification of stages (or apneaic periods) using one-versus-rest approach. In order to determine RR-intervals, an R-peak detection algorithm was executed. Illanes-Manriquez and Zhang [16] developed this algorithm in which R-peak was detected with an indicator that took into account the amplitude and the curvature of the ECG signal to distinguish the R-waves from the other waves of the ECG signal. Once the R-peaks were determined, RR intervals were computed. If an RR-interval value was less than 500 or greater than 1500 milliseconds, it was excluded from the analysis.

The features selected for the classification efforts were derived from the RR-interval values of each epoch. They were the median, inter-quartile range, and mean absolute deviation values. The median is the RR interval value for which half of the values are higher, half lower. The Inter-Quartile-Range (IQR) is the difference between 75^th ^and 25^th ^percentiles of the RR interval value distribution. The Mean Absolute Deviation (MAD) is the mean of absolute values obtained by the subtraction of the mean RR interval values from all the RR interval values in an epoch, i.e.,

MAD = mean(abs(RR_vector − mean(RR_vector))

The purpose of the selection of these features was to exclude the outliers from the analysis. Features like mean, standard deviation, and range are affected by the outliers, and thus classification performance deteriorates when these features are included in the analysis. The performances of each of these features were also tested and found to yield lower classification accuracy rates. No features extracted from the ECG waveform were used, because the aim here was to be as simple and accurate as possible by just working with the RR-intervals.

Using these features, we have designed classifiers to predict sleep stage and apneaic epochs. We have used and compared the performances of the following classification methods: k-nearest-neighbor, quadratic discriminant analysis, and support vector machines. While using these classification methods, our labeling approach was in the form of one-versus-rest. For example, in wake epoch prediction, the epochs with wake stage formed class 1 and all other stages class 2. Similarly, for REM epoch prediction, the epochs with REM stage formed class 1 and all other stages class 2, and so on. In OSA prediction, feature vectors corresponding to OSA and non-OSA epochs were labeled as class 1 and 2, respectively. To be more specific on the feature selection procedure, again, with the possible 6 features mentioned above we formed the feature vectors for each class, for example, median values coming from class 1 to one vector and class 2 to the other vector, and performed two-sample t-test on these vectors. As a result of the t-test, p-values for each subject were computed. This was repeated for each 2-class comparison. The average of p-values indicated that the median, iqr, and mad were more discriminative than the other three.

### Classification Methods

Classification considers a set of samples **x **(feature vectors) of an object or event, each of which has a known class label [17]. This set is referred to as the training set. If we assume that the feature vectors **x **are d-by-1 column vectors and there are n samples in the training set, then the training set will constitute d-by-n data matrix. The problem is then to build appropriate models (or classifiers) using the training data to be able to make predictions about the class of new samples. In this study, the use of three classification (also known as *supervised learning*) methods in sleep staging and apneaic epoch detection was investigated.

### k-Nearest-Neighbor

The k-Nearest-Neighbor (kNN) is a nonparametric pattern classification approach, which has been used in many different applications in science and engineering as a benchmark classifier because of its relatively robust performance. It is a simple technique, in which the classification of a feature vector **x **is performed by assignment of **x **to the class that is most frequently encountered among the k nearest samples. In other words, the test vector **x **is thought to be at the center of a sphere whose radius is grown until it encloses k samples from the training set, hence the name "k-nearest-neighbor". The label of the most frequent samples is assigned to the test vector [17]. This classifier relies on a metric or a distance function among the patterns. It is usually the Euclidean distance, which is also the metric used in this study. Other metrics preferred in the literature are the Minkowski (a special form of this metric is the Manhattan distance) and the Tanimoto metric [17]. The k is a user-defined integer, which is an odd number in two-class cases. We experimented with different k values and *k *= 5 turned out to be the best choice in our application.

This approach can be viewed as an attempt to estimate the *posterior *probabilities *P*(*c_i _***|x**) (given a feature vector **x**, probability that it belongs to the *i*^th ^class, denoted by *C_i _*) from neighboring k training samples.

### Quadratic Discriminant Analysis

In Quadratic Discriminant Analysis (QDA), it is assumed that the class-conditional probability density functions (PDFs), *P*(**x|***c_i_*) , are in the form of d-dimensional multivariate normal (Gaussian) distributions [17]:

(1) $$\text{P}\left(x|c_{i}\right)=\frac{1}{{\left(2\pi \right)}^{d/2}|\Sigma_{i}{|}^{1/2}}\exp\left[-\frac{1}{2}{\left(x-m_{i}\right)}^{T}\Sigma_{i}^{-1}\left(x-m_{i}\right)\right].$$

Here, *i *is the class index, **m***_i _*and **Σ***_i _*are the d-by-1 mean vector and the d-by-d covariance matrix for class *i*, | | and ( )*^T ^*are determinant and transpose operators respectively. In our case, d (the number of features) is 3, since we have chosen to work with 3 RR-interval based features. In QDA, mean vectors and covariance matrices, which are assumed to be different for each class, are estimated using the maximum likelihood approach. For minimum-error-rate classification, we need to assign **x **to the class with the highest posterior probability [17]. Using Bayes' formula for conditional expectations, we can express the posterior probability *P*(*c_i_***|x**) as:

(2) $$\text{P}(c_{i}|x)=\frac{\text{P}(c_{i})\text{P}(x|c_{i})}{\text{P}(x)},$$

where **P**(*c_i_*)is the prior probability of class *i*, which simply corresponds the prevalence of class *i *in nature, **P(x) **is called the evidence, which is nothing but the probability of coming across the sample **x **in the feature space. We note that, while comparing posterior probabilities, the evidence factor **P(x) **can be ignored in Eq. (2), as it is independent of the class index *i*. Then, the class conditional PDF, ***P***(**x**|*c_i_*), which is also referred to as the *likelihood *in the literature, scaled by the class prior probability, P(**x **| *c_i_*) , that is the numerator in Eq. (2), will correctly reflect the posterior probability. When implementing the QDA classifier, usually **P**(*c_i_*)**P**(**x **| *c_i_*) term is not calculated directly, instead its natural logarithm, referred as the discriminant function, is used:

(3) $$\ln\left[\text{P}\left(c_{i}\right)\text{P}\left(x|c_{i}\right)\right]=\ln\text{P}\left(c_{i}\right)-\frac{\text{d}}{2}\ln\left(2\pi \right)-\frac{1}{2}{\left(x-m_{i}\right)}^{T}\Sigma_{i}^{-1}\left(x-m_{i}\right)-\frac{1}{2}\ln\left|\Sigma_{i}\right|$$

The $\frac{\text{d}}{2}$In(2*π *) term can be dropped as it is independent of *i*, the class index. We note that the discriminant functions given by Eq. (3) are quadratic in **x**, hence the name quadratic discriminant analysis.

If one has the knowledge of the prior probabilities, P(*c_i _*) , from past experience for instance, then they can be used directly in Eq. (3). In this study, we have assigned the above-mentioned percentages (see Table 1) of one sleep stage as the probability of occurrence of that specific stage P(*c*_1 _) and the probability of the occurrence of other stages as 1 - P(*c*_1 _). For a test feature vector **x**, if ln[**P**(*c*_2_)**P**(x|*c*_2_)] > In[ **P**(*c*_1_)**P**(**x**|*c*_1_)], then **x **is categorized as class 2, *c*_2_, otherwise as class 1, *c*_1_.

### Support Vector Machines

Support vector machines (SVMs) is basically a binary (two category) classifier that relies on nonlinear mapping of the training data to a higher dimension, thus the transformed data can always be separated by a hyper-plane. Each pattern **x **is transformed to another pattern using some suitable kernel function **y **= *f *(**x**). A linear discriminant in the transform **y **space is *g*(**y**) *= ***W***^T ^***y**, where **W **is the weight vector perpendicular to the hyper-plane characterized by *g*(**y**) *= *0. With this form, a linear discriminant function must go through the origin. However, if we augment **W **and **y **with *b *and 1 respectively, then the linear discriminant can have an offset *b*. The idea is to find a separating hyper-plane which ensures *z_k_g*(**y***_k _*) ≥ 1, where *z_k _*= ± 1 is an indicator variable for pattern *k*, according to whether pattern **y***_k _*= *f*(**x***_k _*) is in *c*_1 _or *c*_2_.

In training an SVM, the objective is to find the separating hyper-plane with the largest margin. This guarantees that the classifier will have a superior generalization performance [17]. The distance from any hyper-plane to a transformed pattern **y***_k _*is |*g*(**y***_k _*)|/**||W||**, and there is a positive margin *b*, for which *z_k_g*(**y***_k _*)/**||W|| **≥ *b *. The goal then becomes finding the weight vector **W **that maximizes *b*. Since there may be infinitely many weight vector pointing into same optimal direction of the separating hyper-plane, the constraint *b ***||W|| **= 1 is imposed on **W **for uniqueness. The support vectors are the transformed training patterns for *z_k_g*(**y***_k_*) = 1 (just on the hyper-plane), which carry all the relevant information about the classification problem. A kernel function *f*( ) capable of well separating the data gives rise to a small number of support vectors and low error rate. Therefore, the selection of the kernel function is important. These functions might be polynomials, Gaussians, or other basis functions such as radial basis functions. In our implementation the kernel function was first order polynomial. In the optimization process we used quadratic programming with a method called sequential minimal optimization [18,19]. This method breaks the optimization problem down into sub-problems that may be solved analytically, eliminating the need for a numerical optimization at each step.

### Evaluation of Classification Performance

Given a set of samples we first randomly divide it into two parts as training and test sets. Then the classifier is trained (model is estimated) using the training data and classification accuracy is estimated by verifying the predictions of the trained classifier on the test set. As the given set of samples varies the classification accuracy rate also varies. Therefore, the classification accuracy rate is a random variable as such it has a bias and variance with respect to the actual classification accuracy rate associated with the underlying classification problem [20]. While separating the overall set of samples into training and test sets, if the size of training set increases the bias in the estimation of classification accuracy decreases and the variance increases. Inversely, if the size of test set increases the bias increases and the variance decreases. A common technique to assess classification performance is 10-fold cross-validation (CV). In this approach the overall set of samples is randomly divided into 10 approximately equal and balanced (i.e., the distribution of samples into different classes is similar) parts. Then, each time one of these subsets is excluded from the overall set of samples and used as the test set and the remaining samples are used as the training set. This cycle is repeated over the 10 subsets and the resultant classification accuracy rates are averaged to produce 10-fold CV accuracy rate. For a subject with total number of 800 epochs, for example, partitioning produced 10 subsets with 80 epochs each. Therefore, the training set (720 epochs) and test set (80 epochs) included totally separate sets of data. The 10-fold CV was repeated for each classification method (kNN, QDA, and SVM) on each subject. The classification accuracy here refers to the ratio of correct decisions (i.e., true positives plus true negatives) to the total of number of cases. In some cases it may be more insightful to report the classification performance using two separate indicators, namely sensitivity and specificity, however in our case, false positives and false negatives are equally important in evaluating performance of sleep stage and apneaic epoch determination. We also used Cohen's Kappa index (CKI), which measures agreement between predicted and actual categorizations. The CKI has a correction for agreements that may occur by chance [21].

## Results

**Figure 1:**
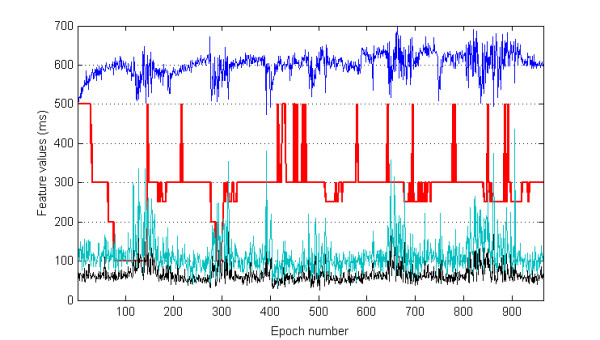
**Simultaneous display of the hypnogram from one healthy subject and the associated values of the features computed from each epoch**. The red lines show wake, NREM 1 to 4, and REM sleep stages with respect to epoch numbers throughout the night's sleep (hypnogram). Because of simultaneous display, on the hypnogram wake and REM are at the levels of 500 and 250 ms, respectively. NREM 1 to 4 stages are shown at 400 to 100 ms levels, respectively. Blue, green, and black graphs indicate median (values are halved for better representation), interquartile range (iqr), and mean absolute deviation (mad) of RR values obtained from each epoch.

**Figure 2:**
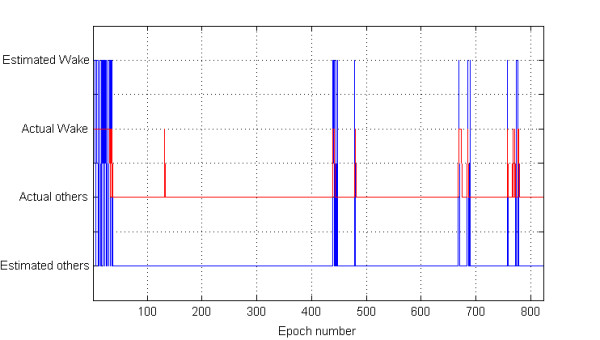
**Classification performance of one healthy subject with respect to the epoch number using QDA as the method of choice**. The red lines show the actual stage as wake or other stage, and blue lines show the estimated stage as wake or other stages. We imposed an offset between the actual and classification results in order to make them easy to differentiate.

**Figure 3:**
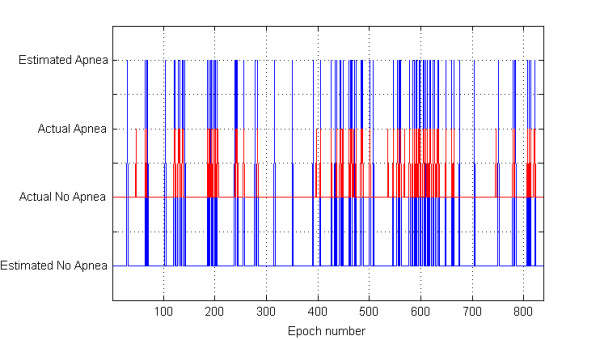
**Classification performance of one subject from OSA group with respect to the epoch number using SVM as the method of choice**. The red and blue lines show the actual and estimated class of an epoch as with apnea or without apnea, respectively. We imposed an offset between the actual situation and classification results in order to make them easy to differentiate.

We have processed a total of 14,219 epochs and selected 13,439 (94.5%) of them for the subsequent classification study. From each selected epoch median, iqr, and mad values were computed from the associated RR values. Figure 1 shows the features and the corresponding sleep stages (hypnogram), simultaneously, with respect to epoch numbers throughout the night's sleep of one representative healthy subject. We note that the variation of the median values on this figure has been shown with half of the values for compactness of the graphic. Figure 2 depicts the comparison of the actual stages and the automatic sleep stage classification results using QDA with respect to epoch numbers from one healthy subject. This figure graphically demonstrates the performance of one of our classification approaches with two states, one of the sleep stages (such as wake) and the other stages. In Figure 3, the performance of the SVM method as the classification approach in detecting the apneaic epochs is shown for one subject from the OSA group. The comparison is represented by the apneaic epochs defined by an expert and those automatically classified.

We report here the percentage of accurate classifications for each of the six sleep stages (wake, NREM 1, 2, 3, 4, and REM) for one-vs-rest scheme. Cohen's Kappa Index (CKI) values are also included in the tables. Tables 2 and 3 present the accuracy levels for healthy and OSA groups, respectively. In addition to the accurate classification percentage of each stage for each group we present the total accuracy indicating the performance obtained throughout the night. As shown in Tables 2 and 3, the sleep stage classification study demonstrated that SVM was the best method for each stage individually and weighted overall. This was true for both healthy subjects and OSA group. QDA and SVM performed similarly well and significantly better than kNN. Because the NREM 2 stage covers the 60 or 70% of the night sleep, the accuracy levels decreased from 80-90%'s (for other stages) to 60-70%. Overall results indicate that out of 4 stages 3 were classified correctly using only three heart-rate-related features. When CKI value approaches to 1, the performance of one particular approach is more valuable and less prone to be by chance. Most of the values obtained in this study are close to 1 except for the case with NREM 2 stage.

**Table 2:** The average performances of kNN, QDA, and SVM classification methods in sleep stage classification on healthy subjects

**Classification**	**Sleep Stages**						**Total**
		
**Method**	**Wake**	**NREM1**	**NREM2**	**NREM3**	**NREM4**	**REM**	**Accuracy**
kNN	322/350	105/109	2159/3773	352/390	775/943	655/836	4414/6407
	= 92%	= 97%	= 58.2%	= 90.3%	= 82.2%	= 78.4%	= 68.9%
	CKI = 0.95	CKI = 0.98	CKI = 0.55	CKI = 0.95	CKI = 0.90	CKI = 0.91	
QDA	332/350	106/109	2253/3773	366/390	821/943	695/836	4581/6407
	= 94.9%	= 97.9%	= 59.7%	= 93.8%	= 87.1%	= 83.2%	= 71.5%
	CKI = 0.97	CKI = 0.98	CKI = 0.56	CKI = 0.96	CKI = 0.94	CKI = 0.95	
SVM	334/350	107/109	2328/3773	368/390	824/943	709/836	4684/6407
	= 95.6%	= 98.5%	= 61.8%	= 94.3%	= 87.4%	= 84.9%	= 73.1%
	CKI = 0.98	CKI = 0.99	CKI = 0.59	CKI = 0.98	CKI = 0.95	CKI = 0.95	

The value in each cell shows the number of epochs with accurate estimations divided by the total number of epochs of that specific stage and the corresponding percentage. Cohen's Kappa Index (CKI) is given for each stage and classification method. The last column indicates the total classification accuracy obtained throughout the night for each method.

**Table 3:** The average performances of kNN, QDA, and SVM classification methods in sleep stage classification on OSA group.

**Classification **	**Sleep Stages**						**Total**
		
**Method**	**Wake**	**NREM1**	**NREM2**	**NREM3**	**NREM4**	**REM**	**Accuracy**
kNN	817/1022	307/333	3124/4975	152/159	71/75	465/538	4957/7102
	= 80%	= 92.3%	= 62.8%	= 95.5%	= 94.6%	= 86.4%	= 69.8%
	CKI = 0.93	CKI = 0.93	CKI = 0.60	CKI = 0.94	CKI = 0.97	CKI = 0.95	
QDA	878/1022	317/333	3487/4975	154/159	72/75	492/538	5425/7102
	= 85.9%	= 95.3%	= 70.1%	= 97.2%	= 96.8%	= 91.4%	= 76.4%
	CKI = 0.97	CKI = 0.95	CKI = 0.65	CKI = 0.98	CKI = 0.98	CKI = 0.97	
SVM	883/1022	318/333	3517/4975	155/159	72/75	494/538	5461/7102
	= 86.4%	= 95.5%	= 70.7%	= 97.6%	= 97.0%	= 91.9%	= 76.9%
	CKI = 0.98	CKI = 0.98	CKI = 0.72	CKI = 0.98	CKI = 0.98	CKI = 0.97	

The representation is similar to that of Table 2.

Table 4 shows the results of the OSA detection study. A similar observation with the sleep stage study was that the QDA and SVM outperformed kNN. In five patients the accurate detection rates were over 89% for better performing methods. It is important to note that one OSA patient had neither NREM 3 nor NREM 4, and in five patients NREM 3 was observed while NREM 4 did not exist.

**Table 4:** The average performances of kNN, QDA, and SVM classification methods in apnea detection.

Classification Method	Mean	Worst	Best
kNN	718/904 = 79.5%	615/904 = 68%	808/904 = 89.4%

	CKI = 0.94	CKI = 0.91	CKI = 0.95

QDA	788/904 = 87.2%	687/904 = 76%	854/904 = 94.5%

	CKI = 0.97	CKI = 0.95	CKI = 0.98

SVM	789/904 = 87.3%	683/904 = 75.6%	854/904 = 94.5%

	CKI = 0.98	CKI = 0.95	CKI = 0.98

The values on the "Mean" column are the average percentages of accurate estimations. The "Worst" and "Best" classification accuracies across OSA subjects are also included.

## Discussions and Conclusion

The aim of this study was to investigate the potential use of single-lead ECG recordings in sleep stage classification and obstructive sleep apneaic epoch detection. Visual inspection of the ECG data and RR-interval determination were the basic processing steps. The median, inter-quartile range, and mean absolute deviation values computed from RR-intervals obtained from each epoch served as the features used in the classification procedure. The k-nearest-neighbor, quadratic discriminant analysis, and support vector machines were preferred as the methods of classification. The ECG data came from healthy subjects and OSA patients. Therefore, it was possible to test the features and classifiers using single-lead ECG for both groups.

For proper feature extraction and classification procedures, going over the data from each epoch was critical, and certainly improved the quality of the study. This was also useful in visual inspection of the R-peak detection performance. The algorithm used for this purpose gave highly successful results. In addition to the three-abovementioned features, the mean, standard deviation, and range values computed on each epoch were tested, however, did not yield sufficient accuracy levels. The features based on ECG wave shape, for instance QT or QRS duration, required the automatic delineation of the ECG signal, which is not a trivial task. Especially, determination of the end-of-T-wave is difficult with automatic analysis. We tested a wavelet-based approach for this purpose; however, its performance was not promising, and thus QRS and QT interval values became unreliable for any further classification efforts. That is why we proceeded with the beat-to-beat interval, which is relatively simple.

We examined the performance of three classification methods because each had a different modeling capacity. The kNN uses no modeling at all, whereas the QDA models the data as consisting of groups or classes with multivariate Gaussian distributions, which is somewhat restrictive. After transforming the data to a higher dimensional space, SVM separates transformed data into two categories with an optimal margin. Both in sleep stage classification and OSA detection purposes, the QDA and SVM methods performed similarly well and better than the kNN approach. We should note here that our SVM implementation utilized a simple linear kernel; with more complex kernels it should be possible to further improve the performance of SVM classification.

Out of 6 possible sleep stages, the classification accuracy was over 83% for 5 stages using the QDA and SVM approaches. Interestingly, the performance of classifiers was better for the OSA group than the healthy group. Another note is that using these methods it was possible to determine whether a person is sleeping or not with an accuracy of ~95% (for healthy group) or ~85% (OSA group). Our results signify that OSA detection using the approach proposed here is feasible and can be an alternative to the systems that use oronasal airflow sensors.

A limitation of this study was the number of subjects whose ECG recordings were obtained. The subjects with consistent ECG morphologies and heart rate characteristics were selected. In addition, subjects with cardiac problems were excluded from the study. Moreover, certain epochs with high amplitude artifacts caused by the body movements that dominated the ECG signal were also excluded in the subsequent analysis. Even though the number of subjects was limited, the number of epochs was large enough (over 13,000 epochs) to make statistical comparisons.

In a future study, we will deal with the automatic detection of noisy epochs in terms of signal itself and the computed RR-interval values. Furthermore, because the QDA method requires the knowledge of prior probabilities of each class the author chose here to use the average incidence percentages as the prior probability values, which is different for healthy and OSA groups. The QDA was the method of choice here instead of linear classifier because of its higher modeling capability with two different covariance matrices for each class, in contrast to linear classifier where there is the restriction of identical covariance matrix. We will deal with the automatic detection of noisy epochs in terms of signal itself and the computed RR-interval values, so that visual inspection of each epoch will not be required. Moreover, automatic delineation of Q, S, start-of-T-wave, and end of T-wave fiducials will provide new ECG-based features in the near future.

As for the issue of multi-category classification of sleep stages, kNN and QDA can readily be used in such a classification setting. Whereas for the SVM, we need to resort to either one-versus-one or one-versus-rest methods to convert SVM, which is basically a binary classifier, into a multi-category classifier. Actually, we have already experimented with a multi-category classification approach, namely QDA, but classification accuracies for different sleep stages did not reach to satisfactory levels, e.g. were less than 70%. However, we are confident that with additional simple fiducial-based single-lead ECG features, we will be able to design classifiers for the explicit classification of each sleep stage.

This study in general showed that RR-interval based classification, which requires only one channel ECG, is feasible for sleep stage and apneaic epoch determination and can pave the road for a simple automatic sleep analysis/classification system.

## Competing interests

The authors declare that they have no competing interests.

## Authors' contributions

BY conceived of the study and drafted the manuscript. MHA provided technical support in implementation of classification methods and helped drafting the manuscript. EA performed signal processing and feature extraction on the data. SY and FO conducted sleep scoring and helped interpreting the results. All authors have read and approved the final manuscript.
